# Supplementary material for: Empirical methods for controlling false positives and estimating confidence in ChIP-Seq peaks
Source: BMC Bioinformatics. 2008 Dec 5;9:523. doi: 10.1186/1471-2105-9-523 (PMC2628906; doi:10.1186/1471-2105-9-523)
Supplement: Additional File 1 — A variety of html documents from the USeq web site detailing the available applications, their best usage, output file type descriptions, command line menus, etc. [file 1471-2105-9-523-S1.zip › USeqUserGuides/JavaDoc.zip!/JavaDoc/util/gen/FileSize.html]

FileSize


|  |  |  |
| --- | --- | --- |
| **Overview** \| **Package** \| **Class** \| **Use** \| **Tree** \| **Deprecated** \| **Index** \| **Help** | |  |
| **PREV CLASS**   **NEXT CLASS** | **FRAMES**    **NO FRAMES**     **All Classes** |  |
| SUMMARY: NESTED \| FIELD \| CONSTR \| METHOD | DETAIL: FIELD \| CONSTR \| METHOD |  |


---


## util.gen Class FileSize

```
java.lang.Object
  util.gen.FileSize
```

**All Implemented Interfaces:**: java.lang.Comparable

---

``` public class FileSize extends java.lang.Object implements java.lang.Comparable ```

---

**Constructor Summary**

`FileSize(java.io.File f)`


| **Method Summary** | |
| --- | --- |
| `int` | `compareTo(java.lang.Object obj)` |
| `java.lang.String` | `getName()` |
| `long` | `getSize()` |
| `void` | `setName(java.lang.String name)` |
| `void` | `setSize(long size)` |

**Methods inherited from class java.lang.Object**

`equals, getClass, hashCode, notify, notifyAll, toString, wait, wait, wait`

**Constructor Detail**

### FileSize

```
public FileSize(java.io.File f)
```


**Method Detail**

### compareTo

```
public int compareTo(java.lang.Object obj)
```

:   **Specified by:**: `compareTo` in interface `java.lang.Comparable`

---


### getName

```
public java.lang.String getName()
```

---


### setName

```
public void setName(java.lang.String name)
```

---


### getSize

```
public long getSize()
```

---


### setSize

```
public void setSize(long size)
```


---


|  |  |  |
| --- | --- | --- |
| **Overview** \| **Package** \| **Class** \| **Use** \| **Tree** \| **Deprecated** \| **Index** \| **Help** | |  |
| **PREV CLASS**   **NEXT CLASS** | **FRAMES**    **NO FRAMES**     **All Classes** |  |
| SUMMARY: NESTED \| FIELD \| CONSTR \| METHOD | DETAIL: FIELD \| CONSTR \| METHOD |  |


---
